# The i-Walk Lightweight Assistive Rollator: First Evaluation Study

**DOI:** 10.3389/frobt.2021.677542

**Published:** 2021-09-08

**Authors:** George Moustris, Nikolaos Kardaris, Antigoni Tsiami, Georgia Chalvatzaki, Petros Koutras, Athanasios Dometios, Paris Oikonomou, Costas Tzafestas, Petros Maragos, Eleni Efthimiou, Xanthi Papageorgiou, Stavroula-Evita Fotinea, Yiannis Koumpouros, Anna Vacalopoulou, Effie Papageorgiou, Alexandra Karavasili, Foteini Koureta, Dimitris Dimou, Alexandros Nikolakakis, Konstantinos Karaiskos, Panagiotis Mavridis

**Affiliations:** ^1^ School of Electrical and Computer Engineering, National Technical University of Athens (NTUA), Athens, Greece; ^2^ Embodied Interaction and Robotics Department, Institute for Language and Speech Processing, ATHENA RC, Maroussi, Greece; ^3^ University of West Attica, Athens, Greece; ^4^ DIAPLASIS Rehabilitation Center, Kalamata, Greece; ^5^ SenseWorks Ltd, Nea Smyrni, Greece

**Keywords:** assistive robotics, intelligent systems, robotic rollator, multimodal systems, elderly, movement disorders, clinical validation, human-robot interaction

## Abstract

Robots can play a significant role as assistive devices for people with movement impairment and mild cognitive deficit. In this paper we present an overview of the lightweight i-Walk intelligent robotic rollator, which offers cognitive and mobility assistance to the elderly and to people with light to moderate mobility impairment. The utility, usability, safety and technical performance of the device is investigated through a clinical study, which took place at a rehabilitation center in Greece involving real patients with mild to moderate cognitive and mobility impairment. This first evaluation study comprised a set of scenarios in a number of pre-defined use cases, including physical rehabilitation exercises, as well as mobility and ambulation involved in typical daily living activities of the patients. The design and implementation of this study is discussed in detail, along with the obtained results, which include both an objective and a subjective evaluation of the system operation, based on a set of technical performance measures and a validated questionnaire for the analysis of qualitative data, respectively. The study shows that the technical modules performed satisfactory under real conditions, and that the users generally hold very positive views of the platform, considering it safe and reliable.

## Introduction

Mobility problems, particularly concerning the elderly population, constitute a major issue in our society. According to recent reports, approximately 20% of people aged 70 years or older, and 50% of people aged 85 and over, report difficulties in basic activities of daily living. Mobility disabilities are common and impede many activities important to independent living. A significant proportion of older people have serious mobility problems. About 8% of people aged 75 years are not able to move outdoors without help, and the percentage increases to 28% at the age of 85. The corresponding percentages in relation to the ability to move indoors are about 5 and 14%, respectively (Heikkinen, et al., 2004). Furthermore, current demographics show that the elderly population (aged over 65) in industrialized countries shows a constant increase. In EU, the rising life expectancy is calculated to bring about an increase of 40% of the population aged 80 and over, in the period 1995–2015, meaning that the aforementioned problems are expected to assume an even greater significance in our society for the years to come.

Mobility is an important activity of the elderly since it promotes physical exercise, independence and self-esteem. Assistive robots can play a significant role since they can incorporate features such as posture support and stability, walking assistance, navigation in indoor and outdoor environments, health monitoring and others.

This paper presents an overview of the i-Walk robotic rollator concept, along with the design, setup, and execution of the first clinical investigation study regarding the real-life performance of its technical modules and the perceived satisfaction of the users regarding its safety, mobility, functionality and usefulness.

The i-Walk rollator is a robotic assistive device, designed in different versions to support a wide range of motor and cognitive assistance functionalities in various use cases. The paper starts by describing, in *Overview of the i-Walk Lightweight Rollator*, the main functionalities and overall architecture of the lightweight version of the device, as it was used in the study presented in this paper. The system integrates a set of perception, navigation and user-robot interaction modules, designed in such a way as to enable real-time operation to support the envisaged user-assistance functionalities. The paper then focuses on describing in detail the first evaluation study, its design and implementation in a real clinical setting, as well as the results obtained. The study comprised a set of scenarios in a number of pre-defined use cases, which include physical rehabilitation exercises, as well as mobility and ambulation involved in typical daily living activities of the patients. These scenarios, together with the study design user groups and inclusion criteria, are fully described in *Materials and Methods*. *Results* then presents and analyses the obtained results, which include: a) objective evaluation of specific system modules, based on a set of technical performance measures, and b) subjective evaluation of specific system functionalities, using a validated questionnaire for the analysis of qualitative data. The study results and findings are further discussed in *Discussion*, and conclusive statements together with future work directions are provided in *Conclusion and Future Work*.

## Overview of the i-Walk Lightweight Rollator

The i-Walk concept concerns the research and development of two intelligent robotic rollators incorporating multimodal human-robot interaction functionalities, providing ambulatory and cognitive assistance to the elderly, and to people with moderate motor impairment. The key motivation originates from our aspiration to devise intelligent mobile robotic mechanisms which can monitor and understand specific forms of human activity *in-situ*, in order to deduce their needs, particularly regarding mobility and ambulation, while also providing context-aware support and intuitive assistance in domestic and clinical environments. i-Walk incorporates technologies employing multimodal signal acquisition and processing, recognition-monitoring-analysis and prediction of user actions, real-time gait analysis, user-adaptive motion control providing docking and front-following behaviors, navigation in dynamic environments, cognitive assistance, verbal and non-verbal human-computer interaction, a natural avatar-based interface, speech synthesis and recognition, and others. The purpose of the devices is to serve as integrated systems to support the physical rehabilitation of patients and also accommodate the clinical evaluation of their progress, based on objective and quantifiable metrics. The ultimate goal is to personalize a patient’s mobility and cognitive assistance and improve their rehabilitation program.

The platform has two user interfaces, involving two different groups, offering different services to each one; the first group concerns the actual patients (i.e., the primary users of the device). This group receives the aforementioned physical and cognitive rehabilitation assistance, and constitutes the main focus of the study presented in this paper. The scenarios evaluated in herein, resemble common everyday activities of the patients. The second group concerns the clinician/carers of the patients (i.e., the group of secondary users of the platform). For this group, the platform enables them to oversee each patient’s progress by evaluating various pathophysiological aspects such as their gait, exercise performance, walking speed, orientation in space during navigation etc. This information is presented to the carers, who can then adjust and personalize the patient’s rehabilitation program, in order to match their actual capacity. In this study, this interface is not evaluated; instead, we have opted for employing the carers group as a baseline when assessing user performance on the technical modules. Furthermore, their overall subjective assessment of the platform provides valuable insights on its utility and safety, from the point of view of an expert.

The i-Walk project offers two solutions (Moustris et al., 2021); a lightweight one, using a commercially available design, retrofitting it with the appropriate sensors and electronics to provide a subset of the functionalities, and a heavier robotic rollator incorporating a full-scale version of all the envisaged functionalities. The former is intended mainly for home use, having no actuation at all while the latter is a fully-fledged motorized robotic rollator, mainly for clinical environments.

**FIGURE 1:**
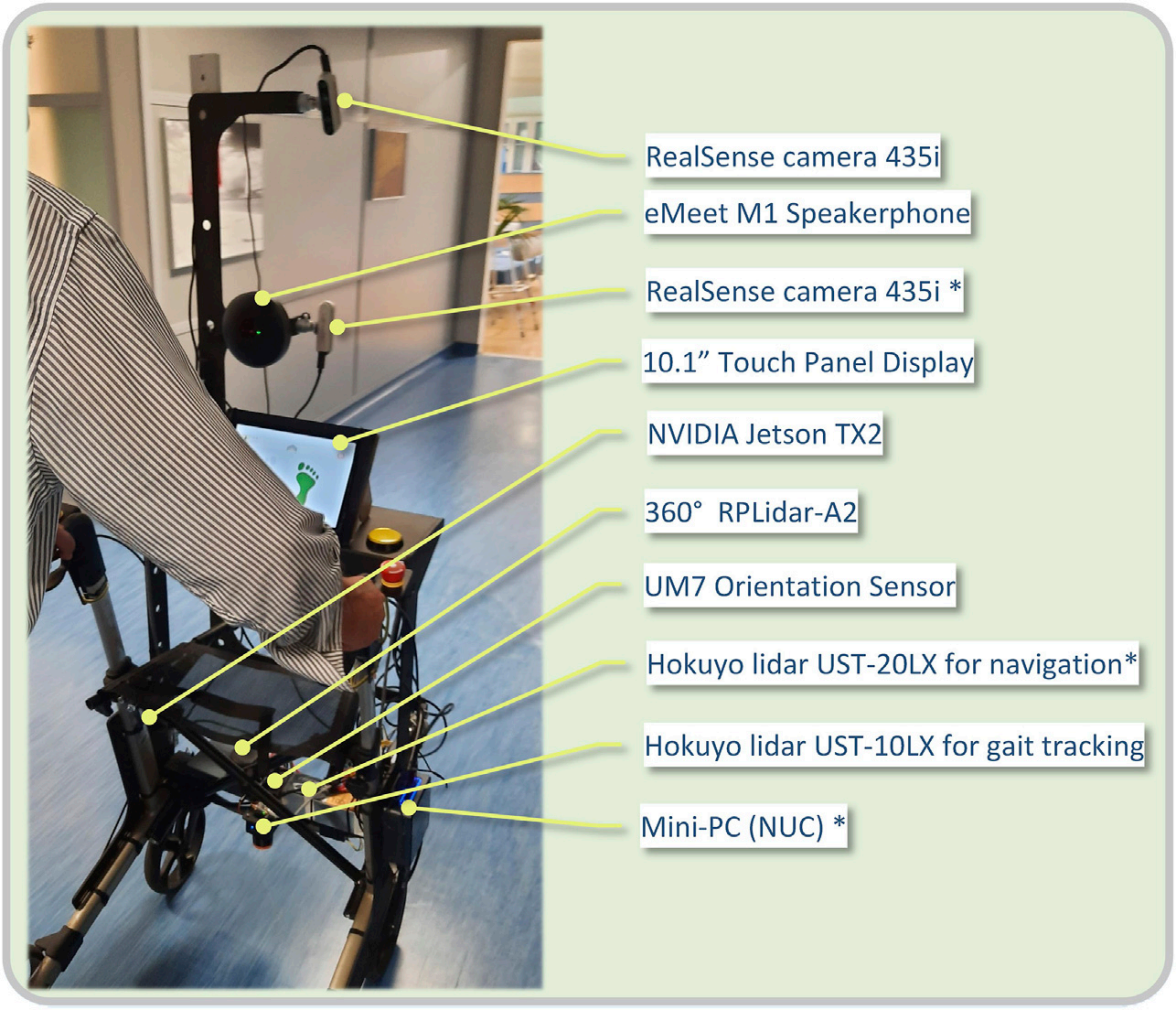
View of a patient with the i-Walk lightweight Rollator. Current hardware configuration is listed on the right.

The version used in the course of the evaluation study presented in this paper, is the lightweight platform (Figure 1), utilizing the hardware listed below:

1. RealSense camera 435i for pose estimation (rear)
2. eMeet M1 Black Conference Speakerphone
3. RealSense camera 435i (front) *
4. 10.1″ Display
5. NVIDIA Jetson TX2
6. 360° RPLidar A2
7. UM7 Orientation Sensor
8. Hokuyo lidar UST-20LX for navigation *
9. Hokuyo lidar UST-10LX for gait tracking
10. Mini-PC (NUC) *

**FIGURE 2:**
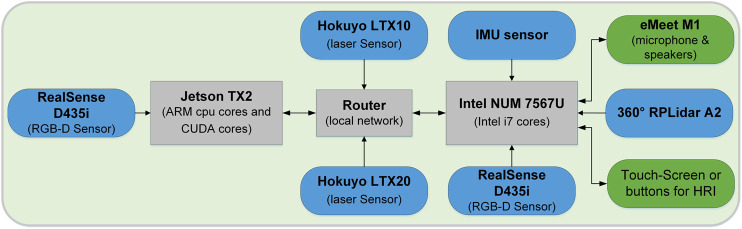
Overview of the i-Walk h/w architecture used in this study.

The items marked with an asterisk (*) are intended for the heavyweight platform. A view of the platform’s hardware architecture is presented in Figure 2.

### Speech Understanding

The platform contains a voice interaction system in order to enable natural communication with the patient, who essentially uses speech to express their intention, i.e. that they wish to walk, or to perform their daily exercise program. The goal of the speech interaction system is the patient’s intention recognition and interaction. It contains an Automatic Speech Recognition (ASR) module, a Natural Language Understanding (NLU) and a dialog management and text-to-speech module. For ASR, speech recorded through a 4-channel microphone array serves as input to the state-of-the-art speech recognition Google speech-to-text API (Google Cloud Speech-to-Text, 2020), and is transformed into text, thus requiring an active internet connection. Subsequently, the transcribed text serves as input to the NLU module, in order to be translated into a human intention. The integrated NLU system has been built with RASA (Bocklisch et al., 2017; Open Source Conversational AI, 2020): A set of predefined intentions, both general purpose and specific to the current application has been designed. The former category includes seven general intents, namely greeting, saying my name, saying goodbye, thanking, affirming, denying, asking to repeat, while the latter one includes seven intents designed for the Human-Robot Interaction: standing up, sitting down, walking, stopping, ending interaction, going to the bathroom, doing exercises. Each intention is associated with various phrases to express this particular intention. For example, users can express their will to stand up by saying “I want to stand up,” “Can you please help me stand up,” or any other variation. A RASA NLU pipeline called *tensorflow embeddings* is then employed to predict the current intention based on the speech transcription. For the dialog part, in order to manage the several intentions and perform the specific actions required or just define what should be played back to the user, RASA Core has been used. Finally, for the actual speech feedback, Google text-to-speech (TTS) in Greek has been employed. All the above mentioned components have been integrated into ROS platform and communicate among them or with other system components, if needed, via ROS messages.

### Visual Action and Gesture Recognition

An integral part of the rollator’s intelligence is its ability to understand patients’ everyday activities i.e. an *action recognition* system. This provides carers and other medical staff with valuable information about the patients’ condition and facilitates overall integration, as other subsystems can have real-time access to the patient’s activity. This is performed in two steps using the back facing RGB-D camera; first the patient’s *3D pose estimation* is calculated, and subsequently *action recognition* is performed using an LSTM based neural network with features computed from the 3D pose (Chalvatzaki et al., 2020). In case the recognized activity is an exercise, the exercise monitoring module presents the corresponding recognition scores, while in case a gesture is detected, the gesture recognition module is triggered.

3D Pose Estimation is performed extracting the 2D body keypoints on the image plane, employing the Open Pose Library (Cao et al., 2017) with the accompanied models trained on large annotated datasets (Andriluka et al., 2014; Lin et al., 2014). The third dimension of the 3D body keypoints is obtained by the corresponding depth maps. Subsequently, given a pair of pixel coordinates for a body joint and the depth value at this pixel, the corresponding 3D joint’s coordinates are calculated through the inverse perspective mapping using the calibration matrix of the camera. For the final human skeleton we discard the keypoints of the face, hands and feet either because in many cases they are not detected, or the corresponding depth values are unreliable. For activity recognition, the 3D locations of the human joints are used as features. We transform the 3D body joint locations which are provided in the camera coordinate system to the body coordinate system with the middle-hip joint as origin. We also normalize them by the length between the left- and right-hip joints (BNORM scheme). In addition, we enhance the pose feature vector with the 3D velocity and acceleration of each joint, computed from the sequence of the normalized 3D joints’ positions.

For human activity recognition we employ a Neural Network architecture based on LSTM units (Hochreiter and Schmidhuber 1997). LSTM constitutes a special kind of recurrent neural networks that can effectively learn long-term dependencies that exist in sequential data, such as human joint trajectories. Our network architecture consists of two LSTM layers stacked on top of each other and a fully connected (FC) layer, followed by softmax activation, to obtain per-class scores. The sequence of the pose features in a temporal window is used as input to the above network. To classify the whole sequence in one of the predefined classes, we apply max pooling on the hidden states of the LSTM network, which, by our in-house experiments, has been shown to yield the best results compared to several other pooling schemes.

### Odometry and Localization

The platform offers location based services, such as pinpointing the user on a map, giving navigating instructions to go from one place to another etc. To this end, i-Walk incorporates an odometry estimation module, along with a localization algorithm which provides a position and orientation estimate on a known map. Since the lightweight version does not include wheel encoders to track the motion of each wheel, we have opted for laser-based odometry through the use of incremental laser scan matching. This algorithm tries to match consecutive laser scans, on the premise that the change is small in each scan, as compared to its previous one, and by tracking their difference, the differential motion of the Lidar sensor can be estimated. By integrating this differential across scans, the total pose estimate can then be computed. Although odometry is reasonably fast (27 Hz in our platform), it accumulates error over time and presents ever growing drift. In our implementation during the evaluation study presented in this paper, we have employed the Canonical Scan Matcher (Censi 2008), through its ROS package (*laser_scan_matcher* package).

Localization refers to estimating the pose of the robot on a map. In our current platform, where a static map is used, an Adaptive Monte Carlo Localization is used (Thrun, Burgard, and Fox 2005), which is a probabilistic localization method in 2D maps involving particle filtering. A direct implementation of this method in ROS is the *amcl* package, which provides an estimate of the robot’s pose fusing information from the front laser scanner and the odometry. The algorithm accounts for the odometry drift over time, and tries to match the laser scans on the map. The amcl is widely adopted and extensively tested on numerous robots.

### Assisted Navigation

The platform incorporates an assisted navigation module, which has been shown to help cognitively impaired people to navigate easier through indoor environments (Koumpouros et al., 2020; Werner et al., 2018). In its current version, the model assumes a known map of the environment and uses predefined paths to guide the patients to places they want to go using audio cues e.g. “walk straight ahead,” “turn right” etc. In general, the walking paths are described by a set of nodes (circles) which have audio tokens associated with them. When the robot enters a node, an audio command is heard, whereas upon exiting, another one is played. Leaving a node renders it obsolete and cannot be re-entered. The position of the robot is provided by the localization module. A view of the reconstructed map of the experimental area, along with the navigation path and nodes, as used in this study, can be seen in Figure 5.

## Materials and Methods

### Clinical Evaluation Scenarios

The following scenarios were designed and implemented in this study, to evaluate the performance of the lightweight i-Walk platform in a set of pre-defined use cases: 1) physical exercises, 2) ambulation with the rollator, 3) assisted navigation, and 4) elevator use. These scenarios were selected to resemble common daily living activities of the patients.

#### Scenario 1: Physical Exercises

In this first scenario, patients are initially seated and perform a series of avatar exercises seen on the rollator screen. Initially, they cross their hands on their chest and perform three torso twists to the left and three to the right. Then, they hold their arms open to the side, and perform three torso twists to the left and to the right. They position their hands on their knees, breath in, extend the arms to the side and return their hands back on the knees while exhaling.

**FIGURE 3:**
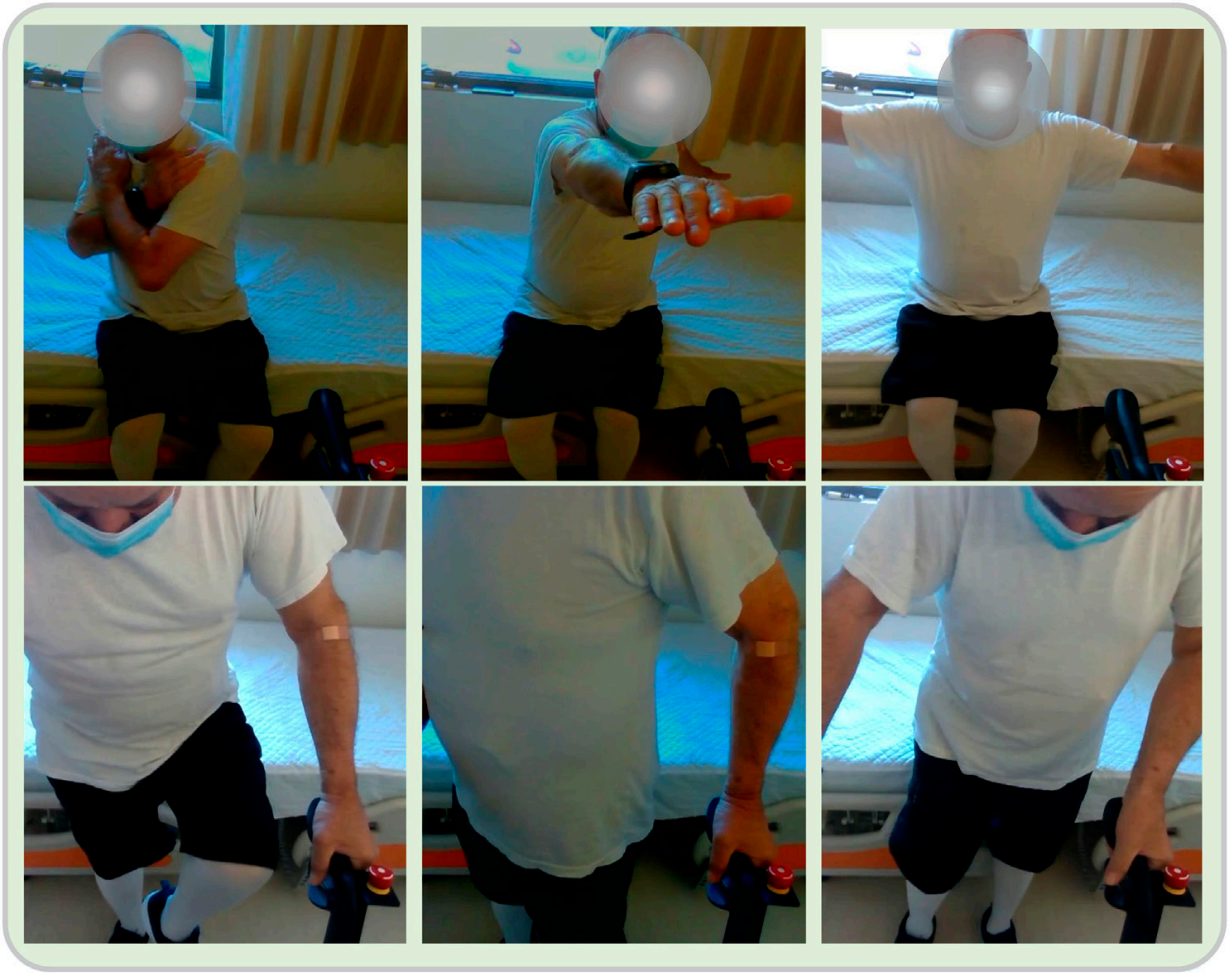
View of the various physical exercises in Scenario 1. Upper Row, Left to Right: The patient perfoms seated torso twists, with the arms crossed on the chest and also extended to the sides. In the third image, the patient extends the arms to the side, breathing in. Lower Row, Left to Right: The patient performs knee-high lifts, torso twists and body transfers.

The patients call the i-Walk platform to come closer, using verbal, or other, communication. After the rollator has approached them, they say the instruction “I want to stand up”. On getting up with the help of the platform, they perform a series of exercises from an upright position, leaning on the platform. Initially, they perform body weight transfers to the right and left, then on-site steps with knee-high lifts and then torso twists left and right from the upright position. Finally, the patients, using verbal commands in physical interaction with the platform, return to their original sitting position uttering the phrase “I want to sit”. They also have the opportunity to stop the exercises by saying “stop” and to inform that the program of exercises was completed with the instruction “we are finished”. A view of various physical exercises involved in Scenario 2 is shown in Figure 3.

#### Scenario 2: Ambulation With the Rollator

**FIGURE 4:**
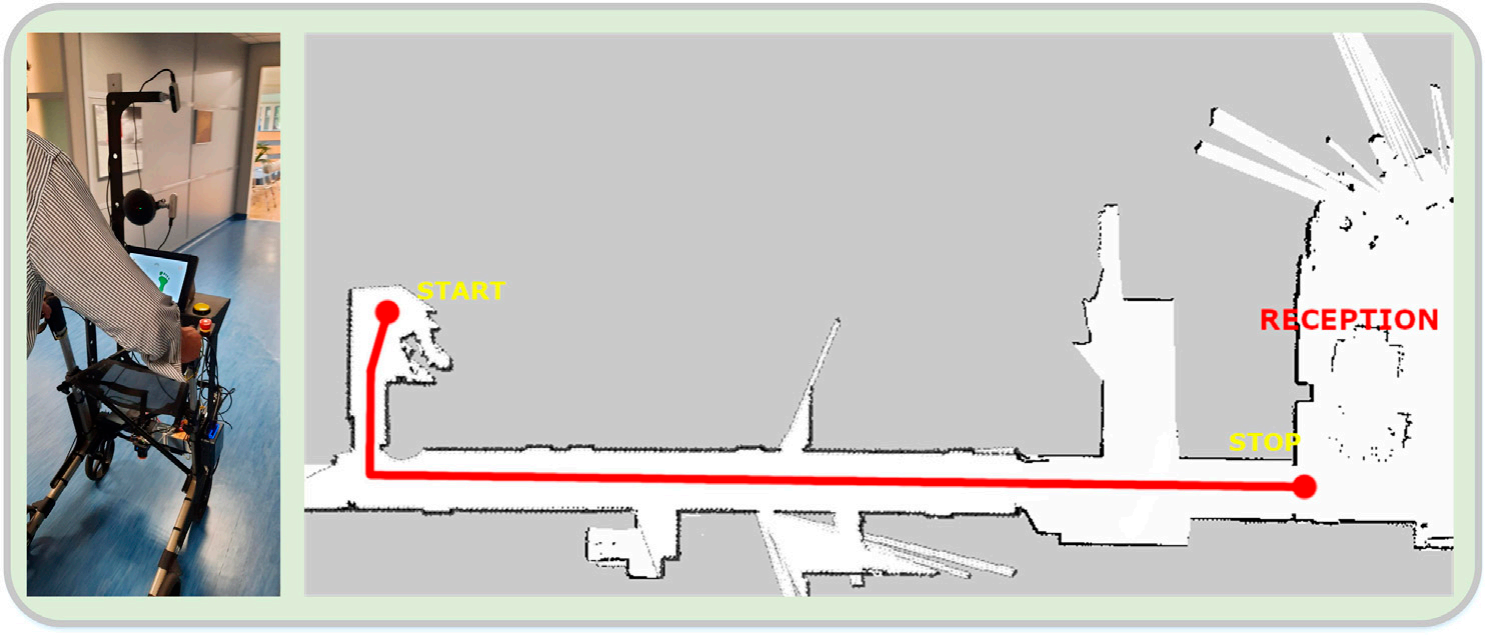
**(A)** View of a patient walking down the corridor with the rollator, in the ambulation scenario. **(B)** Reconstructed map of the experimental area. The intended path is denoted as a thick red line. The patients start from their room, walk down the corridor towards the reception area, and stop at a designated position.

In this scenario, patients start from a sitting position in their room, with the rollator placed in a proper position in front of them. The scenario starts with the patients uttering the phrase “I want to stand,” at which time they grab the handles, bend slightly forwards and proceed to the standing position. The platform recognizes the body posture and gives the appropriate instruction to correct it and achieve good balance. Patients then begin to walk out of the room and into the corridor, following a steady pace dictated using audio and visual instructions by the platform. At the end of the corridor the patients stop, approach a chair by taking steps backward until the posterior surface of their lower limbs comes in contact with it, at which time they set the brakes and sit on the chair (Figure 4).

#### Scenario 3: Assisted Navigation

**FIGURE 5:**
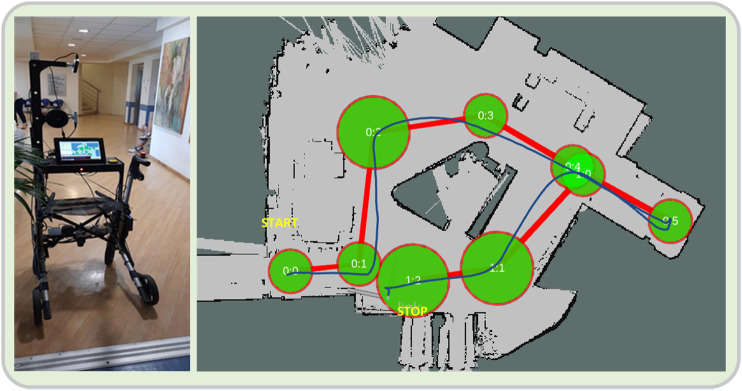
**(A)** Starting position of the assisted navigation scenario. **(B)** Reconstructed map of the experimental area. The navigational nodes are seen as green circles, while the intended path is denoted as a thick red line. A user path from a trial (as estimated and recorded by the localization module) can also be seen (dark blue line).

When this scenario begins, the patients are in a standing position and start to walk, following the audial navigation instructions given by the rollator, regarding the various directions that the patients should take, e.g. “turn right,” “walk straight forward” etc. The instructions were set for a path in the lobby, around the reception desk, where the navigation functionality was evaluated. The users start from a fixed initial position, and try to follow the commands given by the rollator which guide them on a specific path. At the end of each trial, the rollator notifies the users by saying the phrase “you have arrived at your destination,” while they approach a chair by taking steps backward until the posterior surface of their lower limbs comes in contact with it, at which time they set the brakes and sit on the chair (Figure 5).

#### Scenario 4: Elevator Use

**FIGURE 6:**
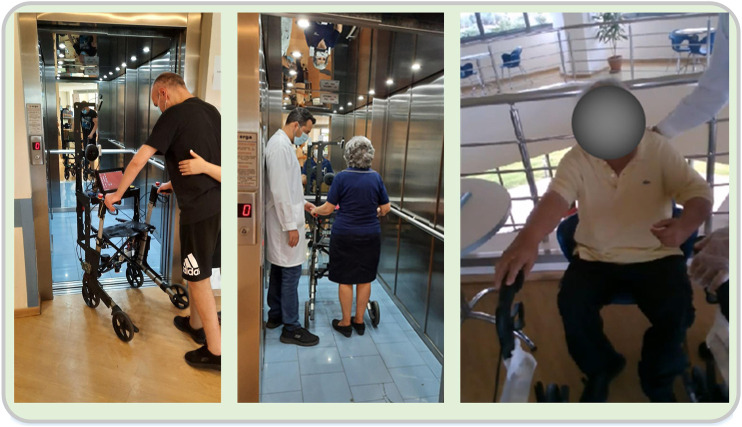
Views of various stages of the “Elevator Use” scenario. The patients are seen entering the elevator, standing to push the floor buttons and sitting on the chair at the final destination.

The fourth scenario involved moving to a specific area using an elevator performing all the necessary maneuvers to enter and exit the elevator. The patients start in a sitting position, and transition to a standing position, remaining there for a few seconds and start walking towards the elevator. After approaching and calling it, they enter inside, perform a turn on the spot and press the appropriate floor button. Following, they take some steps back together with the rollator, and head towards the door. They exit the elevator, move a few meters forward, turn on the post and head back. Upon entering, they turn on the spot in order to press the floor button, take some steps back together with the rollator, and head towards the door. They exit the elevator and move towards a chair positioned next to the door. They approach the chair by taking steps backwards until the posterior surface of their lower limbs comes in contact with it, at which time they set the brakes and sit on the chair (Figure 6).

### Study Population Demographics and Inclusion Criteria

The study population consists of two distinct cohorts; the *patients* and the *carers.* The two cohorts interact with the rollator using different interfaces, and for different purposes. The *patients* receive the primary clinical assistive services of the i-Walk platform, while the *carers* use the platform in an executive way, overviewing and determining the patient activity. The *patients* cohort consists of *N* = 22 individuals (12 women aged 56–89 years and 10 men aged 47–82 years) while the *carers* cohort consists of *N =* 10 therapists (1 nurse, 5 physiotherapists, 1 occupational therapist, 2 speech therapists and 1 psychologist), all in good health.

The selection criteria for the patients included the 5 Times Sit to Stand Test (5TSTS), the 4 m Gait Speed Test (4MGST) and the Mini Mental State Examination (MMSE). Patients with severe cognitive impairment (MMSE < 17) were excluded from the study (Creavin et al., 2016). For the 5TSTS, the patients performed five sit-to-stand transfers, sitting on a standard height chair (43–45 cm) with their time being recorded. Patients who were either unable to stand or needed more than 16.7 s to perform the test, were excluded from the study in accordance with standard practice (de Melo et al., 2019; Shea et al., 2018; Trommelen et al., 2015). For the 4MGST, the patients were asked to walk for 4 m, recording their time. The average walking velocity is a diagnostic marker for instability and reduced functional ability, as well as a predictor for overall health. Following previous similar studies (Koumpouros et al., 2017; Geravand et al., 2017) patients with an average walking speed less than 0.6 m/s were excluded from the study. The inclusion/exclusion criteria are succinctly presented in Table 1.

**TABLE 1:** Patient inclusion/exclusion criteria.

MMSE criteria		
Categories	Degree of impairment	inclusion/exclusion
Category 1	No cognitive impairment (MMSE ≥26–30)	include
Category 2	Mild to moderate cognitive impairment (MMSE 17–25)	include
Category 3	Severe cognitive impairment (MMSE <17)	exclude
**Gait Speed Test criteria**		
Category 1	No or mild mobility impairment (no use of assistive devices, maximum unassisted walking speed >0.6 m/s)	exclude
Category 2	Moderate mobility impairment (use of assistive devices, maximum unassisted walking speed ≤0.6 m/s)	include
Category 3	Severe mobility impairment (unable to use assistive devices, unable to walk)	exclude
**Chair Stand Test criteria**		
Category 1	No or mild impairment (patient can sit and stand unassisted on a standard height chair, 5TSTS ≤16.7 s)	exclude
Category 2	Moderate impairment (patient unable to sit and stand unassisted on a standard height chair or able to stand only from adjustable height chair, 5TSTS >16.7 s)	include
Category 3	Severe impairment (unable to move unassisted, unable to stand from adjustable height chair)	exclude

The patients also presented a range of pathologies; stroke, myopathy, multiple sclerosis, cervical myelopathy, cerebral palsy, rheumatoid arthritis, lower limb fractures and spinal diseases. The mobility aids they used were walkers, rollators, single and four-point gait crutches, as well as underarm crutches. Of the participants in the patients cohort, 54.5% had a high risk of falling (POMA <19) while 45.5% had a medium risk (19 < POMA <24). In addition, 13.6% of them were confined to a wheelchair (0 < BERG <20), 68.2% could walk with some assistance (21 < BERG <40), and 18.2% were able to walk independently (41 < BERG <56). Summary demographics for the two groups are presented in Table 2.

**TABLE 2:** Summary demographics for the two cohorts.

Variables	Patients (N = 22)	Carers (N = 10)
**Age**		
mean ± SD	72.4 (12.0)	31 (2.5)
(minimum, maximum)	(47–89)	(26–35)
**Sex**		
Male	10 (45.5%)	3 (30%)
Female	12 (55.5%)	7 (70%)
**Clinical Test Scores**		
MMSE	25.3	-
BERG	32	-
TINNETI-POMA	18	-
**Former user**		
Walker	12 (54.5%)	-
Crutches	3 (13.6%)	-
Cane	4 (18.2%)	-
Rollator	1 (4.5%)	-
front wheeled walker	1 (4.5%)	-
Nothing	1 (4.5%)	-

Data are presented as mean ± SD with range (minimum, maximum) for age, mean for clinical test scores and n (%) for other variables.

### Methodology and Outcome Measures

The investigation methodology consists of two branches; the first considers the *objective* evaluation of the performance of specific technical modules used throughout the scenarios. Specifically, the following modules of the i-Walk platform were evaluated:

A. Speech Recognition and Dialog.

B. Action Recognition.

C. Assisted Navigation.

The second branch considers the *subjective* evaluation of various functions of the rollator during the trials, using the PYTHEIA scale. PYTHEIA (Koumpouros et al., 2016) is a valid and reliable scale which has already been tested and validated in several studies and research efforts in the domain of rehabilitation and assistive technologies with or without robotic support (Manon M. Schladen et al., 2020; M. Schladen et al., 2020; Manon M. Schladen et al., 2020; Koumpouros et al., 2020). The instrument is divided into two main parts; Part A is related to the evaluation of the assistive technology as a whole, covering various aspects such as fit and ease of use, while Part B can be used as many times as needed in order to evaluate any individual characteristic of the assistive technology (e.g. autonomous navigation, oral commands, etc.). In this study the following aspects were evaluated:

D. Overall Satisfaction with the i-Walk platform.

E. Satisfaction with the platform regarding the physical exercise functionality.

F. Satisfaction with the platform regarding the mobility functionality.

G. Satisfaction with the platform regarding the navigation and communication functionality.

For both the objective and subjective branches, the two cohorts (patients and carers) performed the same scenarios. Thus the carers interacted with the platform in the exact same way as the patients. This study design choice was adopted in order a) to comparatively evaluate a baseline performance of the various system modules by a group of normal functioning subjects (much like using a control group), and b) most importantly, to collect and comparatively evaluate additional feedback related to the viewpoint of the healthcare professionals assessing the performance and operation of the various system modules and intended functionalities. It should also be noted that healthcare professionals and carers constitute actually the main group of secondary users of the robotic assistive device, who will be assigned the role of continuously monitoring the efficacy and safety of its operation, and assessing the effect of the different assistive functionalities on the patients (i.e. the primary users of the device).

To quantify the performance of the technical modules, the appropriate evaluation metrics were selected. For the *Speech Recognition* module, two widely used speech recognition performance metrics were employed: the SCOR (Sentence Correct) metric, and the WCOR (Word correct) metric. The first, SCOR, refers to the percentage of the correctly recognized sentences, while the WCOR to the percentage of the correctly recognized words. For the Dialog module, the Intention Correct metric was employed. The latter is of greatest interest, because it is directly connected to the patient-robot interaction quality. It essentially refers to the percentage of the correctly recognized intentions from the Natural Language Understanding module of the dialog system. For example, a patient may express the intention of performing exercises with various different phrases. The Intention Correct measures the effectiveness of the dialog system to correctly recognize the user’s intention, regardless of the phrase they have used.

For the *Action Recognition* module, we used a predefined set of nine actions which can be recognized by the system. These include generic actions (seated, walking, etc), as well as rehabilitation exercises. We consider that an action is classified correctly if at least 70% of the windows that fall within the temporal limits of the action are correctly classified. To recognize actions in a continuous video stream we employ a non-overlapping temporal sliding window with approximately 1 s duration. The outcome measure of this module is the recognition accuracy of the actions.

The outcome measures for the objective evaluation of the technical modules are presented in Table 3:

**TABLE 3:** Outcome measures for the Objective Evaluation.

Technical module A: Voice recognition	
**Outcome measure**	**Details**
Sentence Correct -SCOR (%)	The percentage of correctly recognized sentences
Word Correct - WCOR (%)	The percentage of correctly recognized words
Intention Correct (%)	The percentage of correctly classified intentions, that refers to the dialog system
**Technical Module B: Action Recognition**	
**Outcome Measure**	**Details**
Recognition Accuracy (%)	The percentage of correctly recognized user actions from the system’s action vocabulary
**Technical Module C: Assisted Navigation**	
**Outcome Measure**	**Details**
Path Completion Time - T (sec)	The time each user takes to go from the starting position to the ending position
Number of Stops - K	The number of the time intervals where the user has a velocity less than 0.1 m/s, for at least 1 s
Total Stop Time - T_stop_ (sec)	The sum of all time intervals where the user has a velocity less than 0.1 m/s, for at least 1 s
Walking Distance—S (m)	The geometrical distance a user travels to go from the starting position to the ending position
Walking Velocity - V_m_ (m/sec)	The average user velocity calculated as the Walking Distance over the Total Walking Time. The Total Walking Time is computed as the sum of all time intervals in which the user has a velocity ≥0.1 m/s

For the subjective validation, the outcome measures reflect the PYTHEIA scale and are essentially identical to it. These are presented in Table 4.

**TABLE 4:** Outcome measures for the Subjective Evaluation.

Outcome measure	Details
Overall Satisfaction (0–5)	Average of the PYTHEIA Part A questionnaire in 6-point Likert Scale
Physical Exercise Satisfaction (0–5)	Satisfaction with the physical exercise functionality. Average of the PYTHEIA Part B questionnaire in 6-point Likert Scale
Mobility Satisfaction (0–5)	Satisfaction with the mobility functionality. Average of the PYTHEIA Part B questionnaire in 6-point Likert Scale
Navigation & Communication Satisfaction (0–5)	Satisfaction with the navigation and communication functionality. Average of the PYTHEIA Part B questionnaire in 6-point Likert Scale

All measures are evaluated in a 6-point Likert Scale, and range from: 0-N/A, 1-Not at all satisfied, to 5-Extremely satisfied.

## Results

### Objective Evaluation Results

**FIGURE 7:**
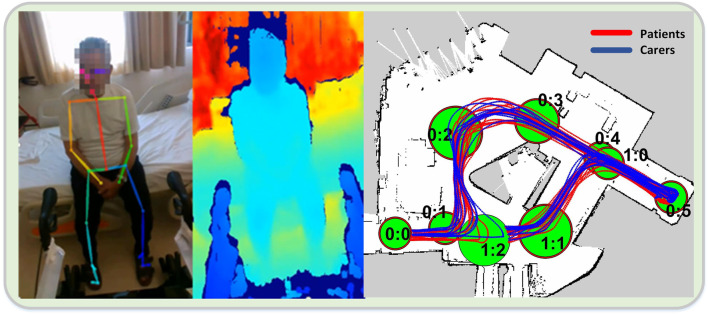
2D pose **(A)** and corresponding depth map **(B)**, in the action recognition module during a trial. **(C)** Patient and carer paths in the assisted navigation trials.

For the evaluation of the technical modules, the patients and the carers performed the four scenarios in succession, i.e., first the physical exercise scenario, then the ambulation scenario, followed by the assisted navigation scenario and ending with the elevator use scenario. Each user performed each scenario exactly once. Overall, there were N = 32 individuals in the two cohorts (22 patients and 10 carers), leading to a total of T = 32 trials. The speech recognition and the action recognition modules were used during the first scenario, while the assisted navigation module was deployed in the third. Thus, only the aforementioned two scenarios contributed to the objective evaluation branch (Figure 7).

From the 32 trials, 32 were evaluated in the speech recognition and dialog module, 22 in the action recognition module (only the patient cohort) and 24 in the assisted navigation module (15 patients and 9 carers). The data streams from all the onboard sensors were recorded in ROS rosbag format and analyzed in post processing, to extract meaningful information regarding each module. This analysis was performed using MATLAB, Microsoft Excel and Python.

During the first scenario, for the evaluation of the speech recognition and dialog module, the patients and the carers interacted with the system using free speech. The dialog was led by the platform which was asking them questions, or instructing them to perform an action or press a button. There were no predefined speech sentences or commands. The user uttered responses in free speech, then the phrases were recognized and the speech recognition result was fed to the dialog system, where it was translated to an intention. According to the recognized intention, the appropriate response or action was performed by the system and the user received feedback in the form of speech synthesis.

For the action recognition module, there were nine predefined actions viz. “Seated on a chair or bed,” “Preparing to stand up using the rollator,” “Standing up from a seated position,” “Sitting down from a standing position”, “Walking using the rollator,” “Arms crossed on the chest in seated position,” “Torso turns with arms crossed on the chest,” “Torso turns with arms open on the side in seated position” and “Torso turns in standing position leaning on the rollator”. Each action was performed a predefined number of times, except for “Walking” and “Seated,” for which the number of instances varied among patients. Instances of “Walking” and “Seated” with duration >30 s were split randomly into shorter segments for evaluation, with a total number of 525 action instances being tested.

Regarding the assisted navigation scenario, in order to have a similar starting and stopping point reference across all trials, the actual path that was analyzed in each trial started from the *point of exit of the first node* and ended at the *point of entry of the last node*. It is noted that when we refer to distance, velocity and time of a user in a trial, we are actually referring to the respective quantities of the robot, since the user is walking holding the robot by the handles, and the two are moving in unison as a single body. The estimation of the instantaneous walking velocity was done in post processing in two steps; first the Euler approximation is calculated by taking the difference of two consecutive odometry measurements, divided by their relative time difference. This leads to a very noisy first estimate which is then smoothed out in the second step by using the Robust Locally Weighted Scatterplot Smoothing (RLOWESS) algorithm. The resulting velocity is used as an estimate for the user walking velocity in the calculation of the number of stops.

The aggregate results, averaged over all individuals per cohort, are given in Table 5.

**TABLE 5:** Objective evaluation results for the three technical modules.

Technical module A: Voice recognition		
**Outcome measure**	**Patients**	**Carers**
Sentence Correct -SCOR (%)	64.20 (23.26)	91.33 (15.58)
Word Correct - WCOR (%)	65.88 (23.37)	91.00 (15.78)
Intention Correct (%)	68.00 (22.73)	93.33 (15.28)
**Technical Module B: Action Recognition**		
Recognition Accuracy (%)	55.97 (12.33)	-
**Technical Module C: Assisted Navigation**		
Path Completion Time - T (sec)	115.77 (26.44)	78.10 (5.62)
K = 0	5 (33%)	9 (100%)
Number of Stops K = 1	9 (60%)	0
K = 2	1 (7%)	0
Total Stop Time - T_stop_ (sec)	9.94 (6.54)	0
Walking Distance—S (m)	38.92 (1.04)	39.69 (0.51)
Walking Velocity - V_m_ (m/sec)	0.37 (0.07)	0.51 (0.04)

Data are presented as mean (±SD), except in Number of Stops where it is the number of individuals (cohort percentage).

### Subjective Evaluation Results

The subjective evaluation was performed using the PYTHEIA structured questionnaire, following the execution of all four scenarios from the two cohorts. Overall, there were 32 trials, the same with the objective evaluation. Statistical analysis was performed using the IBM SPSS Statistics for Windows, version 25.0 (IBM Corp., Armonk, NY, United States) software package. The normality of the data was first checked with a Kolmogorov-Smirnov test. As no normal distribution was found, the non-parametric Mann-Whitney *U* test was then applied. Data processing showed convergence of views between patients and carers on most points/questions.

Regarding the evaluation of the *overall functionality* (PYTHEIA Part A), the average scores of the two groups are 4.1 for the patients, and 4.29 for the carers. Their difference was statistically insignificant (*p* > 0.05). The only discrepancies were found in the satisfaction of the two cohorts regarding i-Walk’s contribution to the improvement of everyday life, and of the weight of the platform.

Specifically, for the question,

Part A - Q2: Rate your satisfaction with the supporting device and the services provided in relation to its contribution to the improvement of your everyday life.

Patients rated i-Walk statistically significantly lower than carers (mean value 3.86 vs 4.6, *p* = 0.041 < 0.05), while for the following, Part A- Q8: Rate your satisfaction with the supporting device and the services provided in relation to the weight.

Even though the patients rated the platform higher relative to the carers (mean value 4.86 vs 4.5, *p* = 0.048 < 0.05), one can consider that the difference is marginally significant and that the views essentially converge.

Regarding the evaluation of *individual functionalities* (PYTHEIA Part B), both groups rated the device very high to excellent (rating > 4 out of 5). Specifically, the following results were obtained:

• For the *physical exercise* functionality, performed with the use of the i-Walk assistive rollator, the average satisfaction of the patients/carers cohorts was found to be 4.7 vs. 4.58 (p > 0.05).
• For the *mobility* functionality, the two groups presented diverging opinions in two, out of five, questions, viz.
○ Part B- Q3: Rate your satisfaction with the specific feature of your assistive device in relation to how safe/secure it is.

In this item, the average patient score was 5.0 vs 4.6 for the carers. This difference was highly statistically significant (*p* = 0.002 < 0.05).

The second questions was,

○ Part B- Q4: Rate your satisfaction with the specific feature of your assistive device in relation to its reliability (i.e. whether it applies always correctly).

In this item the patient cohort responded with an average score of 4.81 vs. 4.4 for the carer cohort. However, even though the opinions are marginally divergent, statistical significance was not very strong (*p* = 0.044) and can thus be contended that the two views are convergent.

• For the *navigation and communication* functionality, different views were upheld by the two cohorts in one of the five questions, specifically in Question 4 (see *Part B-Q4* above). The scores in this question were 4.85 vs. 4.0 for the patients/carers groups, respectively.

**FIGURE 8:**
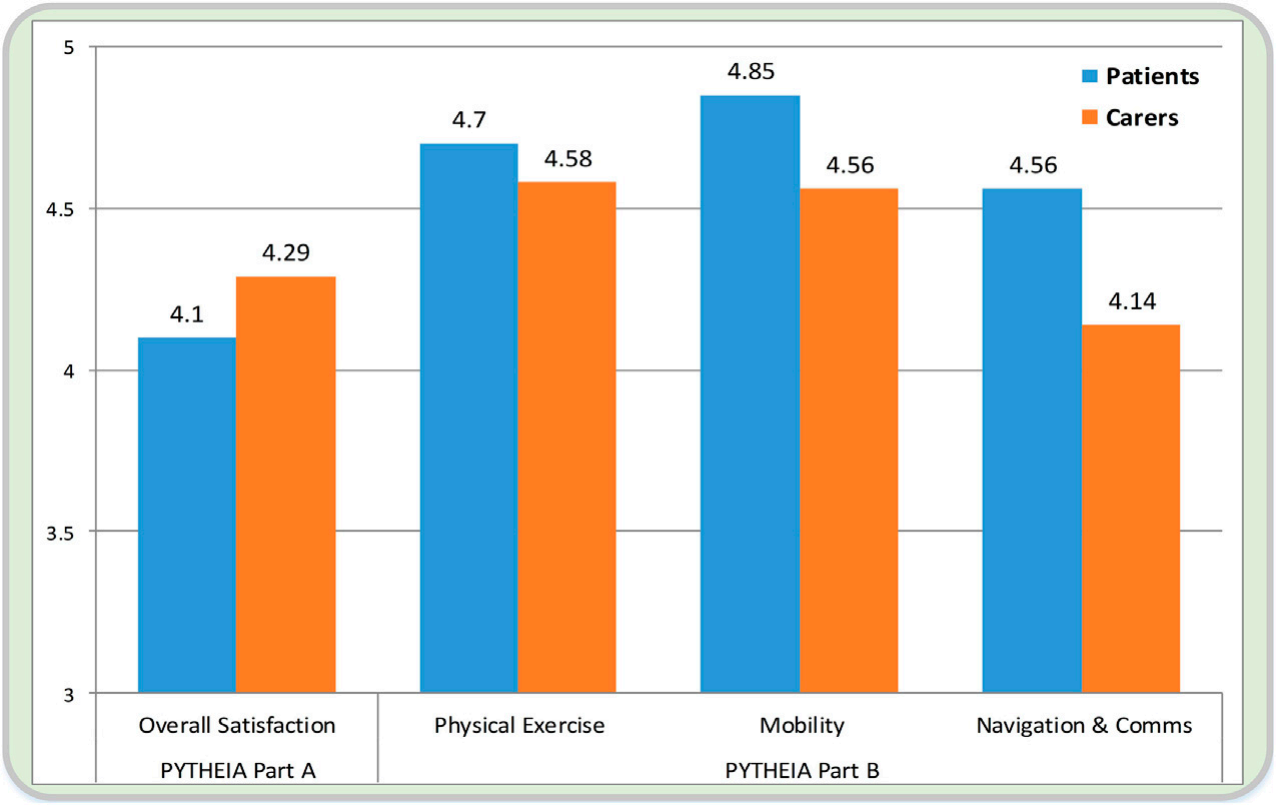
Presentation of the subjective evaluation results between the two cohorts. On the left, the overall satisfaction with the i-Walk platform is seen (average score of the PYTHEIA Part A questionnaire), while on the right, the evaluation of the three individual functionalities is seen (average score of the PYTHEIA Part B questionnaire).

The subjective evaluation results are succinctly presented in Figure 8.

It was also examined whether there was a correlation between fall risk (POMA), reliance on motion assistance (BERG), cognitive deficit (MMSE) and gender, with patient satisfaction for i-Walk. The PYTHEIA Overall Satisfaction score, due to being normally distributed, was tested with the *t*-test to correlate patient satisfaction with MMSE, POMA, gender, whereas the ANOVA method was used to test the correlation with the BERG score. The test showed that there was a marginal difference for patients with a high risk of falling versus patients with a medium risk (median POMA scores 14.95 vs 8.96, *p* = 0.043). Also, the average satisfaction value of patients without cognitive deficit was 3.88 compared to 4.41 for those with cognitive deficit. Finally, the gender and the BERG score of the patients do not seem to affect the satisfaction of the participants at all.

## Discussion

From the objective evaluation results, we can generally see a differentiation between the two cohorts, in the speech recognition and assisted navigation modules. In the former, a noticeable difference can be observed, which mostly relates to patients’ deficits, i.e., cognitive impairment or speech pathologies, as well as to their age. The voice in an older population is usually not as clear as in a younger one, which impedes speech recognition. Additionally, the patients present a large variety of phrases for expressing an intention, due to different local dialects, idiomatic language or by employing indirect ways to express themselves, which could not be totally predicted beforehand. However, in many cases of wrong speech recognition, the final intention prediction was correct, which implies that the dialog system performs quite robustly.

In the assisted navigation scenario, the first positive observation that can be made, when comparing the results of the two groups, relates to the Walking Distance measure. We see that both groups travel the same distance on average, which implies that they tend to walk on the same paths and thus follow the navigation instructions correctly. This finding is also confirmed by observing the results depicted in Figure 7 (right), where a view of all trial paths is provided. However, a clear discrepancy of the two groups is evident in all other output measures. In particular, the patients’ completion time is 50% longer than the carers’, while their average walking velocity is almost 30% slower. This result can of course be directly attributed to the patients’ mobility impairment. Furthermore, at first glance there is a striking inter-cohort variability regarding the number of stops. While the carers do not stop at all during this scenario, most of the patients (67%) stop at least once, raising concerns about the effect of the module on their cognitive load during the trial. A more detailed analysis, however, showed that all the patients stopped at the node *0:5*, where they had to perform a U-turn on the spot. Thus, this apparent stop was due to their lower walking velocity and general impaired ambulation capability, and not to disorientation or confusion. As a whole, the assisted navigation module was able to perform successfully on all trials since all individuals reached the terminal node and passed through all the predefined ones with the correct order. The overall high satisfaction score (4.56) of the patient group in the subjective evaluation regarding the navigation and communication functionality, further corroborates the perceived positive effect of the module.

Considerable intra-cohort variability can also be observed in the patient group regarding the action recognition accuracy (standard deviation ∼26%). This reflects the patients’ diverse mental or physical condition which, in several cases, hindered their ability to perform the exercises correctly, as shown by various related works as well (Rodomagoulakis et al., 2016; Werner et al., 2020). Moreover, the biometrics of each patient have a large impact on the system’s performance, as some users largely fell outside the camera’s field of view. Overall, results show that our approach provides an effective way to recognize elderly users’ actions, indicating the need for a more user-specific setup.

The subjective evaluation results show high satisfaction scores with all three functionalities from both groups, and more so from the patients, who are their primary intended users (scores >4.5). Of great importance is also the fact that both groups considered the platform very safe and reliable whilst their overall satisfaction is high (score >4). It is also interesting to highlight the fact that higher satisfaction scores correlate positively with more severe pathologies, showing that the assistive functionalities of the i-Walk platform have more perceived impact on the patients who are in need the most.

## Conclusion and Future Work

In this paper we presented an overview of the lightweight i-Walk platform, which incorporates multimodal human-robot interaction functionalities, providing ambulatory and cognitive assistance to the elderly, as well as to people with moderate motor impairment. The performance of various technical modules, as well as the perceived subjective satisfaction of actual users with the platform, was investigated in a clinical setting. We described in detail the various evaluation scenarios, output measures and investigation methodology used to assess the various aspects of the device. Results showed that the technical modules performed satisfactory under real conditions with actual patients, and that the users generally hold very positive views of the platform, considering it safe and reliable. The investigation also revealed the special conditions, which can have a negative impact on the performance of some functionalities, for example the use of idiomatic speech from the patients, which must be taken into account in the next design iteration of the i-Walk, tailoring it to the specific population. Future work will involve deployment and clinical validation of the full-scale motorized version of the i-Walk platform, integrating additional modules and assistive functionalities.

## Data Availability

The raw data of the study presented in this article cannot be made publicly available, since they are covered by GDPR and Ethics Approval Guidelines. Anonymized processed data can potentially be shared upon request under controlled access conditions.
